# Caring for a child with a life limiting condition: The experiences of nurses in an intellectual disability service provider

**DOI:** 10.1177/17446295211018588

**Published:** 2021-07-06

**Authors:** Eilis O Connor, Yvonne Corcoran

**Affiliations:** Dublin City University, Ireland; Dublin City University, Ireland

**Keywords:** children, intellectual disability, life limiting, nursing, qualitative research

## Abstract

This study elicited the experiences of nurses caring for children with life-limiting conditions and their family, within a community based intellectual disability service. A qualitative descriptive research approach was adopted where purposeful sampling recruited 10 participants. Data was collected using one to one semi-structured interviews and was subsequently analysed using qualitative thematic content analysis. The findings identified a range of complexities unique to the care of children with life-limiting conditions in the intellectual disability setting. From the findings, it is clear that this is a population of highly skilled nurses who work in a challenging and complex area of practice. Further supports are required in order to meet the practice needs and support the emotional needs of this population of nurses. In doing so, high quality practice within the area will be promoted, thereby ensuring high quality care for the children and families within the disability service.

## Introduction

Globally, it is estimated that there are 21 million children with a life-limiting condition (LLC) (Marston et al., 2018). In Ireland, the Health Service Executive (2020) estimates that there are 8,311 children with a life-limiting condition, approximately 19% (1,579 children) of whom are categorised as being either unstable (15%), deteriorating (2.2%) or dying in any one year. The International Children’s Palliative Care Network define LLCs as conditions for which there is no cure and death is inevitable, either in childhood or early adulthood and where some life-limiting illnesses progress quickly and others may cause a slow deterioration over many years (ICPCN, 2020). From an intellectual disability perspective, Feudtner et al. (2011) estimate that almost 50% of children who have been diagnosed with a LLC also have some degree of cognitive impairment. Thus, a significant percentage of children with LLCs and profound intellectual disabilities are also in receipt of intellectual disability services. Children with both a LLC and a profound intellectual disability often have significant and complex care needs. Care requirements are diverse and dynamic but can typically include subspecialist care, frequent hospitalisations and daily use of medical equipment (Whiting, 2014). This directly affects service requirements for children with LLCs in receipt of intellectual disability services and in turn influences care delivery. Nurses working in this sector now routinely participate in highly skilled and advanced medical interventions for the children in their care.

Children with LLCs in receipt of intellectual disability services represent a subset of an already nuanced population. Over the past number of years, service requirement for children with LLCs have been receiving increased recognition, in part due to an increase in the awareness of the needs of this group of children and their families as well as an increase in prevalence (Quinn and Bailey, 2011). Advancements in medical and technological care have resulted in an increased survival rate of children with LLCs, which in turn has resulted in children surviving longer with increasingly complex health needs (Duc et al., 2017). According to Law et al. (2011) not only has the service requirement for this population advanced but also the approach to service provision as, due to the technological advancements, children with LLCs are spending less time in hospital and an increasing amount of time being cared for in the community.

In Ireland, the publication of two important policies, *A Palliative Care Needs Assessment for Children* (Irish Hospice Foundation (IHF) 2005) and *Palliative Care for Children with a Life Limiting condition in Ireland: A National Policy* (DOHC, 2009) generated developments in the area of community based paediatric palliative care services but certain limitations still remain at a frontline level. Limitations remain in the areas of integrated care (McConnell et al., 2016), working with families (McCloskey and Taggart, 2010), practice competency (Chong and Abdullah, 2017) and emotional impact (Bergstrasser et al., 2017). A recent operational and governance framework published by the HSE (2020) has identified 14 recommendations which attempt to mitigate existing community based challenges. This proposed model for change focuses around the reorganisation and coordination of practice in an attempt to provide appropriate pathways of care for the duration of the child’s care journey (HSE, 2020). However despite its emphasis on practice coordination, this report fails to mention the role played by the intellectual disability service where multidisciplinary, school, respite and residential services are accessed by children with LLCs and intellectual disabilities.

From an intellectual disability service perspective there is an enhanced level of complexity in the provision of care to children with LLCs. As identified by Duc et al. (2017) complexities such as functional ability and diagnosis further impact upon the care provision for this population. Duc et al. (2017) explain that often children with LLCs have an undiagnosed intellectual disability syndrome, this lack of diagnosis further impacts upon the uncertainties of disease trajectory. Similarly, due to the functional capacity associated with intellectual disabilities the communication, symptom assessment and symptom management practices for this population are further complicated (Duc et al., 2017).

The provision of care to children with LLCs has been identified as an advancing aspect of service provision in Ireland as evident at policy level (DOHC, 2009). Within the practice area, advancements in service provision have logically taken place in parallel to improvements in research availability. However, from an intellectual disability perspective this advancement appears to lag behind as the research availability within the intellectual disability sector is significantly inadequate and has been described by Duc et al. (2017: 1120) as ‘*unchartered territory’*.

## Aim

This study was designed to elicit the experiences of nurses who provide care to children with life limiting conditions and their family, in an intellectual disability service provider. The objectives of this study were to investigate if the challenges/complexities, identified within the literature, are also relevant to the intellectual disability setting, to discover the unique experiences of nurses from an intellectual disability service and to gain an understanding of the needs of nurses within this care area.

## Method

A qualitative descriptive methodology was the research approach used to investigate and report the experiences of this nursing population. The qualitative descriptive methodology provided an effective research approach in meeting the aims of the study by providing a means by which the experiences of the nursing population could be investigated and reported.

### Study population and sampling

A purposeful sampling technique was employed in order to ensure access to the required population. The recruitment site for this study was a community based intellectual disability service provider which provides respite, residential, school and multidisciplinary services to children with intellectual disabilities. Participant recruitment took place at each of the three locations where nurses are employed (respite, residential and school). Each location provides nursing care to children with intellectual disabilities, including children with life-limiting conditions, within the parameters of the ICPCN’s definition as ‘*conditions for which there is no cure and death is inevitable, either in childhood or early adulthood*’ (ICPCN, 2020). The inclusion criteria were registered nurses from all grades who provide care to children with LLCs under the employment of the organisation. Exclusion criteria were nurses who were not directly on the staff teams of the organisation where care was provided to children with LLCs. All nurses who fit the criteria were invited to voluntarily participate in the research study. A ‘Plain Language Statement’ was disseminated to potential participants via gatekeepers and potential participants were provided with the contact details of the researcher. Of the entire population of twenty registered nurses, thirteen registered nurses agreed to participate in the study. Informed consent was obtained from each participant prior to their involvement in the research study.

Unfortunately, due to the outbreak of COVID-19, restrictions were imposed during the data collection process and data collection at the school site was not possible. A final sample of ten participants took part in the research study, five participants from the residential service and five participants from the respite service. At the initial data analysis phase, each participant was assigned a pseudonym. Of the ten participants, five were registered intellectual disability nurses (RNIDs) and five were registered general nurses (RGNs). Four nurses had been qualified for under 10 years with the remaining six nurses having been qualified for over 10 years. Eight out of the ten nurses had not received any specific paediatric palliative care training.

### Data collection and analysis

Data was collected using one to one semi-structured interviews. This flexible method of rich data collection elicits the opinions and experiences of participants where a rich data set is generated (Glasper and Rees, 2017). One to one semi-structured interviews were conducted with ten nurses working in two practice locations: respite and residential. As the residential location was the place of work of the researcher, a research assistant was used to facilitate data collection at this site in order to eliminate the possibility of bias in the data collection process. Interviews lasted between 24 to 41 minutes; five interviews were conducted by the researcher (in the respite service) and five interviews were conducted by a research assistant (in the residential service). The researcher and research assistant met before and after the data collection process and an interview guide was used to ensure consistency in the collection process. Interviews were conducted at locations chosen by the study participants for their convenience. All interviews were audio recorded, all audio recordings were transcribed verbatim by the lead researcher and all data was anonymised. The researcher and research assistant met to ensure verification in meaning in the transcribed data.

Qualitative thematic content analysis was the process used to analyse the data collected in this research study. The undertone of thematic content analysis within the qualitative descriptive methodology provided the researcher with a structured framework for data analysis. Data analysis was undertaken using the Six Phase Process of thematic data analysis, outlined by Braun and Clarke (2006) (Table 1). Thematic analysis provides an accessible, flexible approach that can be modified for the needs of many studies, providing a rich and detailed, yet complex account of data (Braun and Clarke, 2006).

**Table 1.** The 6 phases of thematic data analysis.

Phase 1	Familiarising yourself with your data
Phase 2	Generating initial codes
Phase 3	Searching for themes
Phase 4	Reviewing themes
Phase 5	Defining and naming themes
Phase 6	Producing the report

### Ethical considerations

Ethical approval was sought and granted from the University Ethics Advisory Committee and the organisation at which participation recruitment was conducted. Participation was voluntary and consent for participation was given in an informed consent form and verbally prior to commencing each interview. Participants were informed of their right to withdraw from the study at any stage, without consequence. The Nursing and Midwifery Board of Ireland (NMBI, 2015a) guidance document for the ethical conduct of nursing research was employed at all stages of the research process in order to ensure the highest possible ethical standards.

## Results

Following an in-depth process of analysis, three themes (Table 2) were elicited from the data set. Theme One: ‘Caring for a Child with a LLC in the Community – The Perspective of the Nurse’ reported experiences in the provision of a community based intellectual disability service. Theme Two: ‘The Child with a LLC and their Family – Implications for Nursing Practice’ identified the unique challenges presented when caring for children with both an intellectual disability and a LLC. Theme Three: ‘The Professional and Personal Impact of Caring for a Child with a LLC’ identified nurses experiences which had a profound personal or professional impact on them. The next section will provide a detailed account of each theme.

**Table 2.** Themes and sub-themes.

Theme	Sub-theme
Caring for a Child with a Life Limiting Condition in the Community – The Perspective of the Nurse	• A Community Based Service – Challenges and Rewards • Autonomy and Collaborative Practice
The Child with a Life Limiting Condition and their Family – Implications for Nursing Practice	• The Additional Complexities of Caring for Children with Intellectual Disabilities • Working with Families – Trust, Communication and Impact on Clinical Judgement
The Professional and Personal Impact of Caring for a Child with a Life Limiting Condition	• Training V Experience – Merits of Both • The Emotional Impact of the Role on the Nurse • Stigmatisation of Intellectual Disability Nursing • Positive Affirmation and Rewards of the Role

### Theme one: Caring for a child with a LLC in the community – The perspective of the nurse

Nurses identified both positive and negative aspects of caring for a child with a LLC within a community based intellectual disability service. Challenges were identified in the delivery of a community based service, particularly where nurses worked in the respite service. Specific challenges focused on difficulties in the availability of up to date information and the provision of medications. According to the nurses working within the respite service, much of these challenges could be attributed to the high volume of children in receipt of the service, a reliance on families to provide medication and accurate information on the current care needs of the child and a lack of consistency in the dissemination of information to the nursing team.

“school communicates with families, doctors communicate with families, hospitals communicate with families, so…and nobody really remembers that there’s a little respite service out here”…“so it’s a lot of chasing that up”…“nobody really thinks about us.” (Brid)

Across both the respite and residential locations the most significant challenge identified was in relation to the availability of medical support in the event of a child becoming unwell. Here nurses reported concerns regarding autonomous practice and identified the importance of both clinical judgement and collaborative practice in such situations. This was a significant concern, which was a prominent finding of the study and one which had a significant impact on the professional practice of the nurses.

there’s no support you see…there’s no doctor to call…like, emh, on site, that’s the challenge. (Nuala)

This lack of support resulted in nurses experiencing a sense of isolation in their practice.

in here, your like, on your own…. so you have, your just yourself, ah deciding for everything, it’s not like in the hospital, you have back up. (Paula)

Given the significance of this perceived lack of support and its influence on autonomous practice, the nurses within this study further went on to identify how it impacted upon their professional practice. Nurses identified the importance of clinical judgement within such situations.

your nursing judgement comes in…. amh…when it’s time to ring DDoc…or…you need to bring the child to the GP or ring the ambulance…. Your nursing judgement will like, tell you what to do. (Nuala)

Nurses went on to identify systems in place within the organisation which assist in overcoming the challenges associated with this isolated practice, these included; adherence to guidelines (Nuala), a high level of collaborative practice (Kathleen) and the availability of an on-call manager service (Lill). However, each system was not without its limitations and the perceptions of the merits of each produced a dichotomy of experiences among nurses. For example some nurses had a very positive perception of the on-call manager service where other nurses questioned its worth.

### Theme two: The child with a LLC and their family – Implications for nursing practice

The idiosyncratic aspects of caring for a child with both an intellectual disability and a LLC were identified within the study, namely; the absence of a clear diagnosis and compromised cogitative and communication abilities associated with a child with an intellectual disability. Nurses reported that the absence of a clear diagnosis had implications for both care delivery and access to services for children in their care.

our kids fall through the gaps because, they don’t have they may not have a specific diagnosis that warrants palliative care, but but if you look at them really as a whole they definitely do warrant palliative care. (Helen)

Similarly, nurses in this study identified the cognitive and communication difficulties in this group of children as a challenge when providing care and therefore the importance of knowing the child was key.

especially in the ID [*sic*] sector communication is a big thing, dunno, they can’t tell you that they’re in pain. (Kathleen)

Working with families was identified as a significant area of practice by all participants. The development of an effective relationship with the family was viewed as a meaningful process as the family were described as an important resource in the care of the child.

I feel like they know…. the person more than anybody. (Ann-Marie)

The establishment of trust was reported to be a vital practice in the development of an effective relationship with the family.

they have to really trust you…. so as much as possible you know, you have to keep updating them and you really have to gain their trust. (Paula)

Service specific challenges in fostering such a relationship were identified when nurses in the respite location reported;

we have 60 kids here, it’s tough to build a relationship with 60 families. (Brid)

Similarly, service specific challenges were also reported from the residential setting. Here, the absence of family involvement was identified as a challenge and an event which nurses viewed as upsetting.

unfortunately this is the sad thing here in because most of our kids are ward of court… families are not, not involved…. and that that’s the sad part. (Mary)

The relationship with the family was viewed as an area of practice which had both positive and negative implications for nursing practice. Considerable challenges were experienced by nurses when the families expectation/opinion differed from the service capability, most significantly when the family members opinion differed from the clinical judgement of the nurse. In light of the concerns regarding autonomous practice which was also described by participants, this conflict of opinion adds further complexity to the clinical judgement of the nurse.

you’ve a parent on the phone like ‘don’t send them, don’t send them to hospital’ and its hard then… that can be a challenge as well when you’re trying to make a clinical decision for them…. that’s that can be difficult. (Ann-Marie)

Within the context of working with families the most far-reaching theme within the entire data set and a concept identified by all participants is that of empathy. Nurses had an immense empathy for the family and demonstrated an in-depth understanding of the impact of being a parent of a child with a LLC.

parents are I suppose…rightly so, anxious so they’re their advocates and so if I can’t image dunno amh how it must feel someone else caring for you child. (Helen)

### Theme three: The professional and personal impact of caring for a child with a LLC

The value of both training and experience was identified by the nurses who participated in this study as they discussed this intertwined relationship. The weight placed on training versus experience varied between participants however the general consensus is that there is significant value in both.

hands on experience, is… so much more knowledgeable, you remember it more and everything, information is always good to have, so courses are great to do as well. (Brid)

A high level of practice competency was reported by participants however issues accessing appropriate training were outlined. A desire for increased organisational support accessing such supports was expressed. Challenges were identified in the availability of accessible training and this was attributed to the nuanced practice area (children with LLCs) within the intellectual disability service.

Nurses’ emotional attachments to the children and the resulting emotional impacts of their role were identified. Throughout the study nurses referenced the relationship with the child and the subsequent emotional attachment that is intrinsic to the role. *Mary* explained that she had cared for many of the children in the residential setting ‘*since they were babies*’ (these children are now were in their late teens). Understandably the resulting emotional attachment to the child was identified by nurses and the concept of the child and the nurse being ‘*part of a family*’ was repeatedly cited.

residential, you are their family, and they are your family, like you see them every single day of the week nearly. (Brid)

Significantly, the experience of the death of a child had a profound impact on nurses.

yea, you get so attached to them you feel like you’ve lost your own child. (Nuala)

The lack of recognition of the impact that the death of a child has on the nurse was a source of discontentment among participants.

that was probably the upsetting bit through it all…. is that it was ah now, back to normal now, done. (Brid)

Six participants discussed the impact of the death of a child. Of note is the fact that this was an intense and profound topic for these nurses to discuss and this is evidenced in the fact that one participant (Nuala) became upset and tearful when recounting her experiences. Four participants did not raise the topic of the impact of a child’s death and upon analysis it was discovered that these four nurses were working in the service areas for under 2 years and as such had not experienced the death of a child in their care. Nurses reported that this emotional attachment was amplified for nurses in the residential setting as opposed to the respite setting due to the increased level of time spent with the child. In relation to the emotional implications for the nurse, its unavoidable nature is described by *Brid* as being due to the humanity of the service being provided.

“we’re human services, we don’t work behind a computer, go, leave and nothing happens, it’s like it is people’s lives”…“you get experience and all like that, you can, know how you’d adapt to different situations better the next time by learning from them, but we’re not rocks like, we go in we have feelings at the end of the day.”

Other nurses also identified practices which they implemented in order to assist in coping with the emotional impacts of the role. Activities such as reflection (Lill), exercise (Helen), support from family (Kathleen) and support from friends (Ann-Marie) were all identified as external practices which nurses utilised in dealing with the personal impacts of the role. However, the most prevalent source of support identified within the data was the support of the staff team. This team support was considered to be a vital resource in assisting nurses to deal with the emotional impacts of the role.

we got a good team and dunno that keeps us going, we support each other. (Mary)

A perceived lack of organisational support in dealing with the emotional implications of the role was expressed by participants. Nurses identified practices such as debriefing (Ann-Marie) and psychology input (Nuala) as potential organisational interventions which would support them in dealing with the emotional impacts of the role. Appropriate interventions in light of the reported impact of the role.

it changes ya, like it changes how you think. (Ciara)

From the accounts provided by participants it is clear that this is a highly medically skilled nursing population who perform a role that greatly impacts both their personal and professional lives. In light of this, it is disappointing to learn that some nurses reported upon the stigma associated with this nursing discipline.

that stigmatisation oh like ‘you’re an ID [*sic*] nurse, you know you don’t know what hard work is'…. like, drive you mad, drive you mad, because its, its, 24-hour care its constant, its go go go go. (Ann-Marie)

Although this theme was only reported by a small number of participants this is a significant consideration for the profession as it may have a considerable impact upon the professional identity of the nurse.

Despite the wealth of challenges identified and the significant personal impact for the nurse, all participants reported on the positive aspects of the role. Nurses valued positive feedback, which primarily came from positive affirmation from the family and the child.

when they come in, and, they’ve a big hug for ya, you know you’re doing something right. (Kathleen)

The sense of satisfaction in witnessing the progress of the child due to the quality of care and value of the service being provided was also reported to be a positive experience of the role;

“if you see them, like, learn something new, you know”…“your just SO happy”…“I think it’s nice to see them grow”…“how they’ve come on, and especially like one of the boys he only came to us last year and he was so unwell when he came.” (Ann-Marie)

The narrations provided by the nurses in this study identify the fact that the rewarding aspects of the role assist in outweighing its abundant challenges.

working in this area I find, its rewarding, its rewarding as well, it’s hard but it’s rewarding and that keeps you going. (Mary)

## Discussion

In their recent work Duc et al. (2017) suggest that there is a lack of research exploring the interface between quality palliative care and children with intellectual disabilities. Thus the accounts provided by the nurses who participated in this study provide a valuable insight by reporting on the additional complexities experienced by children with both a LLC and an intellectual disability. The findings of the study identify both the unique experiences and challenges, faced by nurses caring for children with LLCs in the intellectual disability sector. These experiences and challenges include autonomous practice, enhanced competency, working with families, emotional impacts, stigma and rewards.

Autonomous practice was a significant challenge for nurses who provided care to children with LLCs in the intellectual disability residential and respite settings. More specifically this related to the availability of medical support for emergency care if a child became unwell. Currently, within the organisation where nurses who participated in this study were recruited, there are three avenues for out of hours support: an on call nurse manager; GP services; and the acute setting (accessed via the emergency department). However, nurses reported that these support systems were often unsuitable and unwieldly and resulted in delays in ensuring timely medical support for the children in their care. This lack of support resulted in nurses experiencing a sense of isolation, additional responsibility and vulnerability that impacted upon their clinical judgement and caused concern regarding professional responsibility. Nurses discussed the importance of working within their scope of practice (NMBI, 2015b) however, the sense of isolation within this nursing population was clearly an issue of great concern for the nurses who participated in this study. Thus, the findings of this study have identified a need for increased collaborative support for nurses caring for children with LLCs in the intellectual disability setting. Although the requirement for increased collaborative practice has been identified within the existing literature (Law et al., 2011) this was in relation to the co-ordination of allied services and not specifically in relation to the co-ordinated medical care of the child. An increased collaborative approach needs to be co-ordinated and involve key stakeholders in the medical care of the child, including nursing teams, clinical nurse specialists, on-call nurse managers, General Practitioners and acute medical teams. Potential solutions for increased collaboration identified in the literature include the development of ‘Advanced Practice Nurses’ (Morgan, 2009) in the intellectual disability setting who would liaise with key stakeholders and external agencies and act as a co-ordination point to enhance collaborative care. This would ensure that all key stakeholders are informed and up to date on the current medical needs of the child. In addition, the development of emergency care guidelines to be implemented in the event of a child becoming unwell would ensure that key stakeholders are well-informed of the current medical needs of the child, thus reducing nurses experiences of isolation and enhancing their clinical judgement.

Nurses also reported how the absence of a clear diagnosis posed challenges for the care of children with LLCs and intellectual disabilities. This lack of diagnosis resulted in complexities regarding care planning due to the uncertainties of disease trajectory. Diagnostic overshadowing was also reported in regard to accessing acute services. Here nurses believed that symptom severity was often attributed to the intellectual disability as opposed to the LLC and as a result nurses felt that this had a negative impact on the child accessing services. Javaid et al. (2019) outline that diagnostic overshadowing is a contributory factor to the health inequalities experienced by individuals with intellectual disabilities as it results in delayed diagnosis and treatment. As a result, children with LLCs in the intellectual disability setting face increased challenges when accessing acute service. A challenge which undoubtably impacts on the practice of the nurse in the intellectual disability setting as they strive to deliver service user centred and best practice care. Similarly, the compromised cogitative and communication abilities associated with the intellectual disability were reported by nurses to be a source of complexity in the identification of symptoms in the child. In this regard nurses identified the importance of knowing the child in order to effectively identify non-verbal cues of discomfort or symptoms of underlying medical concerns.

The nurses who participated in this study reported a high level of competency and only identified limited concerns in this area. This is in contrast to the existing literature where a significant practice competency need was identified for nurses (Chong and Abdullah, 2017). The existing literature is largely based upon the experiences of community nurses, where a significant competency need was identified due to a lack of training and experience specifically relating to the care of children (Law et al., 2011). The increased level of practice competency reported within this study may be attributed to the intellectual disability setting at which the nurses practice. Due to the nature of the intellectual disability service being provided the nurses in this study have regular, consistent contact with the child so continuity of care was possible. This is in contrast to some services reported in the literature where community nurses may have significantly less contact with the child (Neilson et al., 2013). In this study, there was no significant difference in the experiences reported by registered general nurses and registered intellectual disability nurses, who provided care to children with an intellectual disability and LLC.

However, some contradictions were present in the findings of this study. Although participants reported a high level of practice competency, challenges in accessing appropriate training were also reported as a lack of formal training was identified. Nurses reported a lack of organisational encouragement to participate in formal training and described how they had become autonomous in sourcing appropriate training. While informal systems for learning were present within the intellectual disability setting, nurses reported a challenge in accessing training for continued professional development. Similar to the reports of the existing literature, nurses attributed this challenge to the nuanced area of practice (Neilson, 2010; Quinn and Bailey, 2011). The care of children with LLCs represents a significantly low population within the grand scheme of healthcare provision. As such, nurses require increased organisational support in accessing appropriate training. Recently, the Nursing and Midwifery Board of Ireland (2017) recognised the skillset of RNIDs by placing an increased emphasis on clinical skills for undergraduate nurses, however this emphasis needs to progress to the continued professional development of such nurses. Both the existing literature (Neilson et al., 2013) and the findings of this study would suggest that increased integration, at an organisational level, between services (intellectual disability, acute, hospice, community) would assist in the sharing of expert skills and increase the access to appropriate training for nurses caring for children with LLCs. Here organisational collaboration with acute paediatric settings and the acute paediatric hospice setting would provide a pathway for nurses in the intellectual disability setting to access training appropriate to the care of children with LLCs. A mentoring programme would be very beneficial for the dissemination of skill, particularly for newly qualified nurses or nurses new to the practice area. This practice would further support the existing literatures recommendation of the dissemination of expert skills and experience in order to promote a best practice approach (McConnell and Porter, 2017).

The relationship with the family was identified as a source of complexity by nurses within this study. Nurses identified the vital role of the child’s family, within the integrated care team and acknowledged their importance as a source of information and support. All nurses identified the importance of fostering the relationship with the family. The process of the development of ‘*trust’* within the relationship was viewed as an essential practice in both the existing literature (Carter et al., 2012) and the findings of this study. ‘*Respect’* is also a concept identified within the existing literature (Bergstrasser et al., 2017). Although the concept of ‘*respect’* is not explicitly stated by the nurses who participated in this study, the level of empathy reported by these nurses demonstrated their level of respect for the family. The narratives provided by these nurses demonstrated an immense empathy for the family and an in-depth understanding of the impact of being a parent of a child with a LLC.

Nurses reported that considerable challenges and potential conflict was experienced when the family members’ opinion differed from the clinical judgement of the nurse. Conflict, in the context of the relationship with the family, is not a new concept and has been identified within the existing literature. The literature reports that the existence of this conflict may be influenced by the emotional needs of parents in highly stressful situations (McCloskey and Taggart, 2010) which results in a source of stress for nurses (McCloskey and Taggart, 2010). Nurses working with children with LLCs in intellectual disability services are working in an extremely emotional environment, as demonstrated by the narratives of nurses in this study. This conflict coupled with the challenges associated with autonomous practice adds further complexity to the experiences of this group of nurses.

A unique finding of this study, that was reported by nurses in the residential setting, is the absence and lack of involvement of some families which they described as upsetting and the nurses expressed their empathy for children in such situations. It may be considered that such experiences influence the strong emotional attachment to the child, reported by the nurses in this study. This means that nurses in the intellectual disability setting may experience an increased sense of responsibility for the child. Given the fact that the existing literature and policy (DoHC, 2009) is centred around the provision of services not only to the child with a LLC but also their family, the concept of the lack of involvement of the family highlights the uniqueness of the delivery of care for nurses and children in intellectual disability residential settings. This finding is supported by the existing literature as it reports how this nursing role is highly challenging, demanding and requires a high level of emotional involvement often resulting in emotional distress (Bergstrasser et al., 2017). By its nature a residential service involves a child being consistently cared for by the nurse for a prolonged period of time. For some children, the service is their home and there may be little to no family involvement. As a result, not surprisingly, nurses in the residential setting reported how they formed strong emotional bonds with children who may have been in their care for many years. When a child receiving care in the service died, nurses reported how they viewed the death of this child equated to the loss of a family member. The emotional attachment to the children in their care, that nurses in this study reported, suggests that the level of emotional impact may be further exasperated for nurses working in the intellectual disability setting. As such the investigation into the experiences of this nursing population has identified a significant need in the provision of emotional support in order to assist nurses to overcome the emotional burdens of the role. Additionally, the lack of recognition of the impact that the death of a child has on the nurse was also a source of discontentment identified by nurses. Although this lack of recognition was not directly identified in the existing literature it may relate to the level of disenfranchised grief reported by numerous studies (McConnell et al., 2016; Mac Dermott and Keenan, 2016). For the nurses who reported on the death of a child their narratives demonstrated that it undoubtably had a profound impact on them. Both existing literature (Mac Dermott and Keenan, 2016) and the findings of this study identify the increased need for organisational support to meet the emotional needs of nurses caring for children with LLCs. Suggested organisational interventions in the form of debriefing and psychology support were provided by the nurses who participated in this study. As such organisational intervention is required, as a matter of urgency, in order to prepare and support nurses in the emotional impacts which are inherent and inevitable within their role.

A concerning concept, unique to the care of children with LLCs in the intellectual disability setting, was identified by nurses as being the stigmatisation of the intellectual disability nursing discipline. Goffman (1968) describes stigma as a physical or social characteristic that devalues the individual’s social identity and limits their access to social acceptance. Narratives provided by nurses suggested that within the acute setting the discipline is viewed as lesser skilled and the intellectual disability discipline as a lesser demanding environment. This is a very disappointing and concerning finding and somewhat perplexing in light of the extensive narratives provided by nurses which demonstrated both the level of clinical skill within the population and the increased demands of the environment. A further examination of the existing literature uncovered no data relating to the stigmatisation of the intellectual disability nursing discipline. Nonetheless, this finding is a significant consideration for the profession as it may have a considerable impact upon the professional identity of the nurse. As identified by Slattery (2003) stigmatisation has the potential to result in anger and frustration, which in turn has the potential to negatively impact the nurses level of job satisfaction.

Despite the wealth of complexities identified, the findings of this research study concur with existing literature that identifies the many rewarding aspects of caring for a child with a LLC (McConnell and Porter, 2017; Mac Dermott and Keenan, 2016). The relationship with the child was identified as a source of reward and affirmation for nurses. Witnessing the progress of the child due to the quality of care and value of the service being provided gave a sense of satisfaction to nurses. Nurses who participated in this study cited terms such as rewarding and fulfilling when describing their work and through the narratives provided a sense of pride within their role was evident. Both this research study and the existing literature have identified the immense challenges and complexities experienced by nurses caring for children with life LLCs (Quinn and Bailey, 2011; McConnell, Scott and Porter, 2016). However the reported rewards of the role clearly assist, to some degree, in mitigating these challenges as they offer a sense of satisfaction and affirmation for this nursing population. The importance of the rewarding aspects of the role should not be underestimated as they provide a sense of job satisfaction to nurses who participate in a significantly challenging area of care. According to Perley (2016), a lack of job satisfaction is a primary cause of staff turnover in healthcare, thus the rewarding aspects of the role are a significant factor, which will assist staff retention within a population of expert and highly skilled nurses.

## Limitations and future research

During the data collection process, due to events outside of the control of the researcher, data collection from the sample of school nurses was not possible and as such their perspective was lost. On reviewing current literature, the opinions of school nurses concur with the findings of this study. A study undertaken by Kruger et al. (2009) identified a need for improvement in collaborative practice and access to ongoing education and training for school nurses caring for children with LLCs and intellectual disabilities, both key findings of this research study.

Due to the time constraints of the project, sample recruitment was conducted within a single organisation, thus hindering the generalisability of findings. The final sample size of ten nurses was sufficient to meet recommendations (Holloway and Galvin, 2016) for qualitative research. There was only one male participant, which may have had implications for gender bias within the findings.

## Recommendations for practice

A reliance on families to provide medication and accurate information on the current care needs of the child was a challenge identified by nurses within the respite setting. Although Health Passports are in place within the organisation their primary use is to facilitate communication with the acute setting. The adaptation of such passports may be an effective means of increasing the effectiveness of communication with families.

Nurses reported the significant challenges and complexities of autonomous practice and in particular the lack of medical support in the provision of emergency medical care in the event of a child becoming unwell. An increased collaborative approach needs to be co-ordinated and involve key stakeholders in the medical care of the child. The appointment of ‘Advanced Practice Nurses’ (Morgan, 2009) would provide a co-ordination point for services. This would assist in the development of emergency care guidelines and ensure that key stakeholders are well-informed of the current medical needs of the child. As such, in the event that a child becomes unwell the nurse will have access to guidelines which support practice as well as support systems (general practitioner and on-call manager) who are up to date and well-informed.

Nurses require increased organisational support in accessing appropriate training. Increased inter agency collaboration is required at an organisational level in order to source and secure training relevant to the needs of these nurses. A system such as a mentoring programme would also be very beneficial to the dissemination of skill, particularly for newly qualified nurses or nurses new to the practice area.

An organisational level intervention is required, as a matter of urgency, in order to prepare and support nurses with the emotional impacts which are inherent and inevitable within their role. Organisational intervention in the form of debriefing and psychology support (as suggested by participants of this study) is required in order to ensure that the emotional needs of these nurses are being met. This research reported potential stigmatisation of the intellectual disability nursing discipline. This finding is a substantial consideration for the profession as it may have a considerable impact on the professional identity of the nurse, career development and workforce retention. Thus further investigation is warranted within this topic area in order to establish the stigma perceptions, capture the experiences of intellectual disability nurses and ensure the potential for stigma is ameliorated.

## Conclusion

This study has presented valuable information on the unique experiences of a nuanced population of nurses, underrepresented within the existing literature. The development and provision of services for children with LLCs has been identified as an area of practice which is rife with complexity, due mainly to the rarity and uncertainty associated with care provision (Bradford et al., 2012; McNamara-Goodger and Cooke, 2009; Neilson et al., 2013). These complexities are further enhanced when considered from an intellectual disability perspective (Duc et al., 2017). There are a significant percentage of children with LLCs in receipt of intellectual disability services, these children have complex care needs and these needs are often met by nurses in intellectual disability services.

However, this population are not adequately represented within the research and it has been described as ‘*unchartered territory’* (Duc et al., 2017: 1120). Nurses within this population are a source of valuable information and research within this area of practice will ensure that service provision is adequately meeting the needs of both the child and the nurse.

The findings of this study provide important understanding of a range of challenges, across aspects of service provision, that this group of nurses experienced while providing care to a child with a LLC and an intellectual disability. They included isolation in the delivery of a community based service, the additional complexities of caring for a child with both a life-limiting condition and an intellectual disability, working with families and the professional and personal impacts of the role that nurses. The nurses who participated in this study represent a highly skilled population who participate in a significantly challenging and complex area of practice. The narratives provided in this study give a much needed insight into the dedication and commitment of this nuanced population of nurses, as they strive to deliver evidence based, high quality care. The findings reveal their commitment to practice in an area which has an obvious emotional burden on them. As such, it is of significant importance that every effort is made to support these nurses and ensure that organisational interventions are appropriate in meeting both their needs and the needs of the children in their care.
